# Foramen rotundum versus canal of the maxillary nerve

**DOI:** 10.1007/s00276-026-03930-x

**Published:** 2026-07-06

**Authors:** Mugurel Constantin Rusu, Diana Alexandra Bănică, Cătălin Constantin Dumitru, Adelina Maria Jianu, Răzvan Costin Tudose

**Affiliations:** 1 Division of Anatomy, Department 1, Faculty of Dentistry, “Carol Davila” University of Medicine and Pharmacy, 050474 Bucharest, Romania; 2 Department of Anatomy and Embryology, Faculty of Medicine, “Victor Babeș” University of Medicine and Pharmacy, 300041 Timișoara, Romania; 3 Municipal Emergency Clinical Hospital of Timișoara, 300041 Timişoara, Romania

**Keywords:** Foramen rotundum, Maxillary nerve canal, Cone-beam computed tomography, Skull base morphometry, Anatomical variation, Pterygopalatine fossa

## Abstract

**Background:**

The foramen rotundum, located in the greater wing of the sphenoid bone, transmits the maxillary nerve (V2) from the middle cranial fossa to the pterygopalatine fossa. Despite its designation as a foramen, this structure possesses measurable length, suggesting it functions as a canal. While cross-sectional dimensions have been documented, canal length remains underreported. This study aimed to evaluate the morphometric characteristics of the foramen rotundum canal (FRC) using cone-beam computed tomography (CBCT).

**Methods:**

A retrospective study was conducted on CBCT scans of 45 adult patients (27 males, 18 females) acquired using an iCAT system. Multiplanar reconstructions were analysed using Planmeca Romexis software. Canal length was measured bilaterally from the intracranial to extracranial opening. Statistical analysis included paired and independent *t*-tests, Wilcoxon signed-rank and Mann-Whitney *U* tests, with significance set at *p* < 0.05.

**Results:**

Eighty-eight FRC measurements were analysed. Mean canal length was 4.07 ± 1.78 mm (range: 0.75–9.12 mm), with a coefficient of variation of 43.8%. The left side demonstrated significantly longer canals than the right (4.33 ± 1.77 mm vs. 3.81 ± 1.77 mm; *p* = 0.013). Males showed longer canals than females (4.22 ± 1.87 mm vs. 3.84 ± 1.65 mm), though not statistically significant (*p* = 0.325). One case with bilateral accessory foramina rotunda was identified and described separately.

**Conclusions:**

The foramen rotundum demonstrates measurable canal-like dimensions with considerable variability and significant left-sided predominance. These findings support reconceptualising this structure as the FRC, with implications for skull base surgery and trigeminal interventions.

## Introduction

The foramen rotundum (FR), located in the greater wing of the sphenoid bone, transmits the maxillary nerve (V2) from the middle cranial fossa to the pterygopalatine fossa [1, 6, 9, 11, 14, 15, 24]. The FR is oriented more vertically than horizontally [23]. Despite its name, this structure has a measurable length, suggesting it functions as a canal rather than a simple aperture [5]. Although the FR was regarded as a short canal in the antero-medial portion of the greater sphenoidal wing, authors determined its breadths, width and intracranial distance, but not its length [5].

Accurate morphometric data on the FR are clinically relevant for percutaneous trigeminal interventions, endoscopic skull base surgery, and maxillary nerve blocks. Cone-beam computed tomography (CBCT) enables precise visualisation and measurement of such small craniofacial structures using multiplanar reconstruction.

Although previous studies have documented variability in the cross-sectional dimensions of the FR [5], the canal length has received limited attention [14, 15]. Anatomical variants, including absence, duplication (0.64%) and accessory canals, have been sporadically reported and may influence surgical planning and the diagnosis of pathologies such as trigeminal neuralgia (TN) or tumor infiltration [4, 15, 18, 24].

The aim of this study was to measure the length of the foramen rotundum canal (FRC) using CBCT and to assess differences by side and gender. Anatomical variants observed during the investigation are also detailed.

## Materials and methods

### Study design and ethical considerations

This retrospective cross-sectional study evaluated the morphometric characteristics of the foramen rotundum canal using cone-beam computed tomography (CBCT). The study protocol adhered to the principles of the Declaration of Helsinki. CBCT scans were obtained from patients who underwent imaging for diagnostic purposes unrelated to the present study.

### Sample selection

A total of 45 patients (27 males, 18 females) were included in this study. Inclusion criteria were: (1) adult patients (≥ 18 years), (2) CBCT scans with adequate image quality for measurement, and (3) bilateral visualisation of the foramen rotundum region. Exclusion criteria included: (1) presence of pathology affecting the skull base or pterygopalatine fossa, (2) history of trauma or surgery in the maxillofacial region, (3) congenital craniofacial anomalies, and (4) artefacts or insufficient image quality precluding accurate measurements.

### CBCT image acquisition

All CBCT scans were acquired using the iCAT cone-beam computed tomography system (Imaging Sciences International, Hatfield, PA, USA). The scanning parameters included a field of view (FOV) sufficient to capture the skull base bilaterally, with voxel sizes ranging from 0.25 to 0.4 mm to ensure adequate resolution for accurate measurements of the foramen rotundum canal. Patients were positioned with the Frankfort horizontal plane parallel to the floor, and head stabilisation was achieved using the integrated positioning system.

### Image analysis and measurements

CBCT datasets were exported in DICOM format and analysed using Planmeca Romexis software (Planmeca Oy, Helsinki, Finland). Multiplanar reconstruction (MPR) was used to optimise visualisation of the FR in the axial, coronal, and sagittal planes. The length of the FR was measured as the distance from the intracranial opening (middle cranial fossa) to the extracranial opening (pterygopalatine fossa). All measurements were performed bilaterally (right and left) and recorded in millimetres.

To assess measurement reliability, all CBCT datasets were independently evaluated by three observers: one senior anatomist (MCR, > 20 years experience in craniofacial imaging) and two trained junior observers (BDA and RCT). Prior to the study, all observers underwent a calibration session using 10 randomly selected scans not included in the final analysis. Each observer independently measured FRC length bilaterally on all 45 patients, blinded to the others’ results. Measurements were performed at least two weeks apart to minimise recall bias for intraobserver assessment. The FRC was identified on sagittal slices from the middle cranial fossa to the pterygopalatine fossa openings. The measurement axis was aligned with the canal’s longitudinal axis to obtain the actual anatomical length.

### Statistical analysis

Interobserver and intraobserver reliability were evaluated using the intraclass correlation coefficient (ICC) with a two-way random-effects model for absolute agreement, calculated using EViews 12 (IHS Global Inc., Irvine, CA, USA). ICC values were interpreted as: <0.50, poor; 0.50–0.75, moderate; 0.75–0.90, good; >0.90, excellent. Descriptive and inferential statistical analyses were performed in Python 3.x using the SciPy statistical library. Descriptive statistics included the mean, standard deviation (SD), standard error of the mean (SEM), median, range, interquartile range (IQR), 95% confidence interval (CI), and coefficient of variation (CV). The Shapiro-Wilk test was used to assess normality. For normally distributed data, parametric tests were used: a paired *t*-test for side comparisons and an independent *t*-test for gender comparisons. For the gender comparison, the unit of analysis was individual side measurements (53 sides from 27 males, 35 sides from 18 females); readers should note that treating sides from the same individual as independent observations may marginally affect precision of standard errors, and this is acknowledged as a limitation of the present analysis. Non-parametric alternatives (the Wilcoxon signed-rank test and the Mann-Whitney U test) were also applied. Levene’s test was used to assess the equality of variances between groups. Effect sizes were calculated using Cohen’s d. Bilateral asymmetry was quantified as the absolute difference and the relative percentage difference between the right and left sides. Statistical significance was set at *p* < 0.05.

## Results

### Sample characteristics

A total of 45 patients (27 males, 18 females) were enrolled, yielding a potential pool of 90 side measurements (45 patients × 2 sides). Two individual side measurements were excluded prior to analysis: (1) the right-side measurement of one male patient with bilateral accessory foramina rotunda (Case #1), in whom the accessory variant precluded standard canal length measurement on that side; and (2) the left-side measurement of one female patient with an inferior rotundal canal variant (Case #2), which likewise precluded standard measurement. The remaining 88 measurable foramen rotundum canal (FRC) measurements were analysed (44 right-side and 44 left-side measurements). Because Case #1 contributed only a left-side measurement and Case #2 only a right-side measurement, neither patient could contribute a bilateral pair; consequently, the paired side-comparison analysis was performed on the 43 patients with both measurements available.

### Interobserver and intraobserver reliability

Interobserver agreement among the three raters demonstrated almost perfect reliability, with an intraclass correlation coefficient (ICC) of 0.94 (95% CI: 0.91–0.96) for a two-way random-effects model assessing absolute agreement. The mean absolute difference between observers was 0.31 ± 0.24 mm. Intraobserver reliability, assessed by repeat measurements at a two-week interval on a subset of 20 randomly selected scans, yielded ICC values of 0.96 (MCR), 0.93 (BDA), and 0.92 (RCT), all indicating excellent reproducibility. Given the high concordance, final measurements used for statistical analysis represent the mean of all three observers’ values.

### Overall canal length measurements

**Fig. 1:**
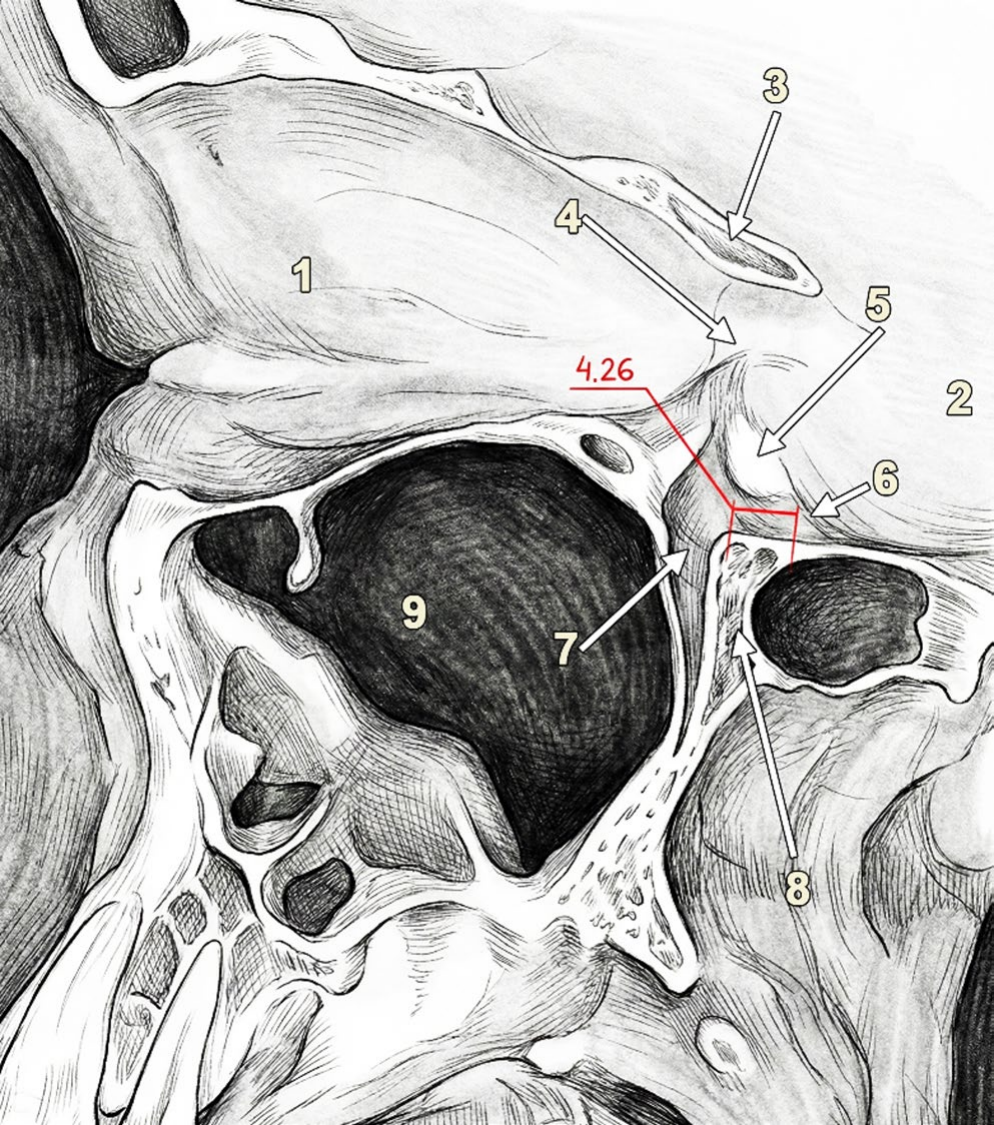
A medium length (4.26 mm) of the foramen rotundum canal is determined on a sagittal slice, on the left side (drawing). Lateral view. (1) orbit; (2) middle cranial fossa; (3) lesser sphenoidal wing; (4) superior orbital fissure; (5) maxillary strut; (6) foramen rotundum canal; (7) pterygopalatine fossa; (8) pterygoid root; (9) maxillary sinus

The mean length of the foramen rotundum canal was 4.07 ± 1.78 mm (mean ± SD), with a median of 3.79 mm. Canal length ranged from 0.75 mm to 9.12 mm, demonstrating substantial anatomical variability (Fig. 1). The 95% confidence interval for the mean was 3.70–4.44 mm. The interquartile range (IQR) was 2.54–5.23 mm, and the coefficient of variation was 43.8%, indicating high inter-individual variability. The Shapiro-Wilk test confirmed normal distribution of the data (*W* = 0.974, *p* = 0.072). Descriptive statistics are presented in Table 1.

**Table 1:** Descriptive statistics for foramen rotundum canal length (mm)

Group	*n*	Mean ± SD	Median	Range
Overall	88	4.07 ± 1.78	3.79	0.75–9.12
Right side	44	3.81 ± 1.77	3.59	0.75–7.52
Left side	44	4.33 ± 1.77	4.50	1.00–9.12
Males	53	4.22 ± 1.87	3.98	0.75–9.12
Females	35	3.84 ± 1.65	3.76	1.75–7.84

SD, standard deviation

### Side comparison

The left side showed longer canal measurements (4.33 ± 1.77 mm) than the right side (3.81 ± 1.77 mm). Paired analysis of 43 subjects with bilateral measurements revealed a mean absolute asymmetry of 1.31 ± 1.04 mm between sides, corresponding to a relative asymmetry of 35.9 ± 29.6%. Both the paired *t*-test (*t* = − 2.214, *p* = 0.032) and the Wilcoxon signed-rank test (*W* = 267.5, *p* = 0.013) demonstrated a statistically significant difference between sides. The effect size was small to medium (Cohen’s d = 0.34). Statistical comparisons are summarised in Table 2.

**Table 2:** Statistical comparisons between groups

Comparison	Test	Statistic	*p*-value
Right versus Left	Paired *t*-test	*t* = − 2.214	0.032*
Right versus Left	Wilcoxon signed-rank	*W* = 267.5	0.013*
Male versus Female	Independent *t*-test	*t* = 0.989	0.325
Male versus Female	Mann-Whitney *U*	*U* = 1045.0	0.318
Normality (overall)	Shapiro-Wilk	*W* = 0.974	0.072
Variance equality	Levene’s test	*F* = 0.487	0.487

*Statistically significant (*p* < 0.05)

### Gender comparison

Males exhibited longer canal measurements (4.22 ± 1.87 mm; *n* = 53 sides from 27 patients) than females (3.84 ± 1.65 mm; *n* = 35 sides from 18 patients; difference = 0.38 mm). However, this difference did not reach statistical significance (independent *t*-test: *t* = 0.989, *p* = 0.325; Mann-Whitney *U* = 1045.0, *p* = 0.318). The effect size was small (Cohen’s *d* = 0.22). Levene’s test confirmed equality of variances between genders (*F* = 0.487, *p* = 0.487). Males demonstrated slightly greater variability (CV = 44.2%) compared to females (CV = 42.9%). Note that the gender comparison was conducted at the side-measurement level; the non-independence of bilateral measurements from the same patient should be considered when interpreting this result (Table 3).

**Table 3:** Canal length stratified by gender and side

Group	*n*	Mean ± SD (mm)	SEM (mm)
Males – Right	26	4.05 ± 1.93	0.38
Males – Left	27	4.39 ± 1.82	0.35
Females – Right	18	3.47 ± 1.50	0.35
Females – Left	17	4.23 ± 1.75	0.42

SD, standard deviation; SEM, standard error of the mean

### Canal length distribution

Canal lengths ranged from 0.75 to 9.12 mm, with a coefficient of variation of 43.8%, indicating high inter-individual variability. The distribution by percentiles was as follows: 5th percentile = 1.75 mm, 10th percentile = 1.96 mm, 25th percentile = 2.54 mm, 50th percentile (median) = 3.79 mm, 75th percentile = 5.23 mm, 90th percentile = 6.61 mm, and 95th percentile = 6.87 mm. The 5th–95th percentile interval (1.75–6.87 mm) may serve as a reference range for distinguishing normal anatomical variation from pathological narrowing in future outcome-based studies.

### Case report #1: bilateral accessory foramina of the foramen rotundum region

During the morphometric study, one male patient was identified with bilateral duplication of the foramen rotundum. His right-side measurement was excluded from the main analysis due to the accessory variant precluding standard canal measurement; his left-side measurement was retained. Because only one side was available for this patient, he was excluded from the paired side-comparison analysis. This case is presented separately due to its rare anatomical findings.

**Fig. 2:**
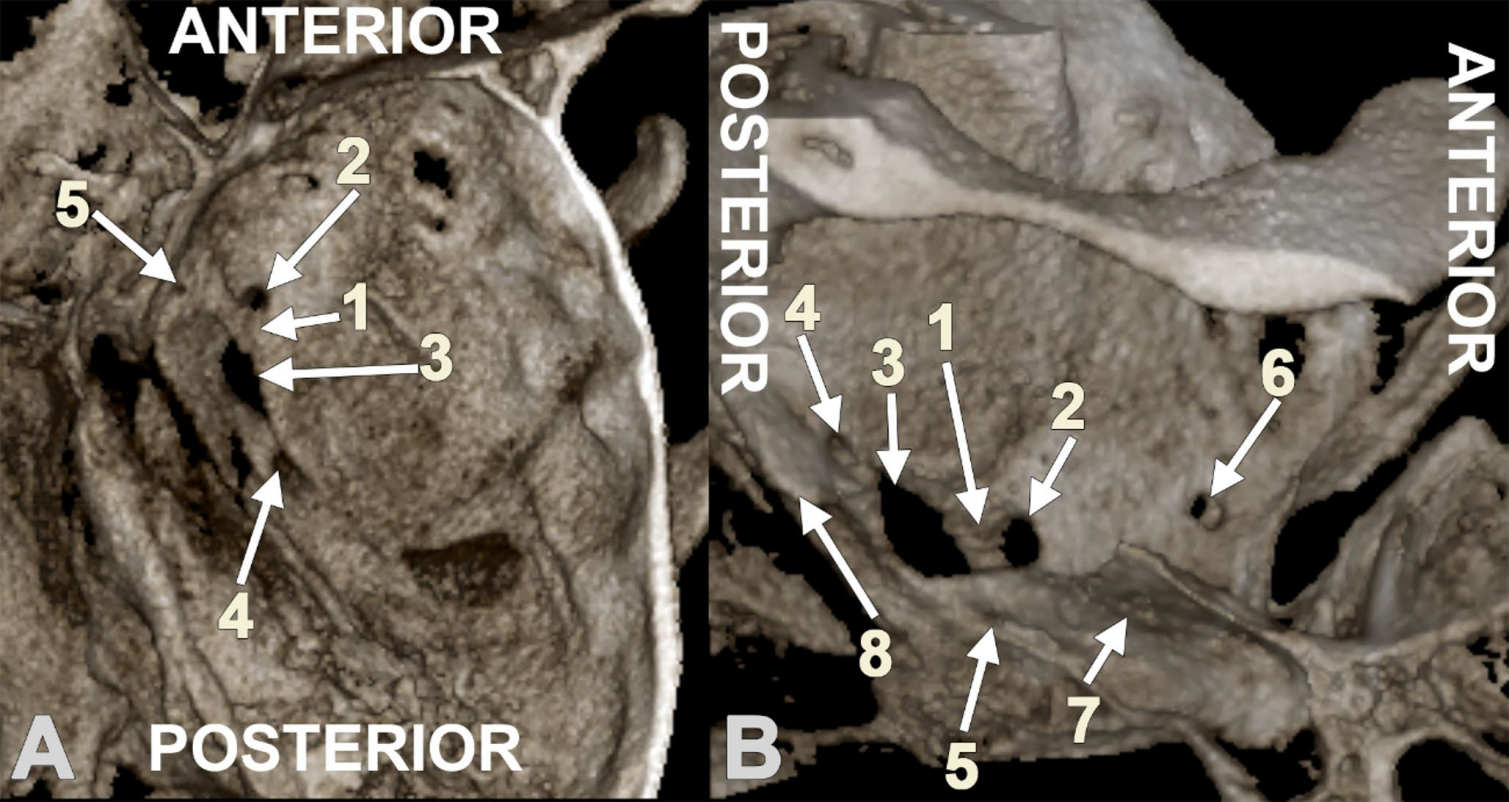
The divided right foramen ovale variant. Three-dimensional volume renderings. **A** Superior (endocranial) view. **B** Inferior (exocranial) view. (1) complete intraforaminal spur; (2) anterior part of the forameu ovale; (3) posterior part of the foramen ovale; (4) foramen spinosum; (5) foramen of Vesalius; (6) accessory foramen rotundum (lateral rotundal canal); (7) lateral pterygoid plate; (8) sphenoidal spine

On coronal multiplanar reconstructions (MPRs), the anatomy of the middle cranial fossa was observed. The sphenoid sinuses were asymmetrical: the left one, postsellar, occupied almost entirely the body of the sphenoid, while the right one was presellar, located in the anterior part of the sphenoid body. On each side, the wings of the sphenoid bone, as well as the plates of the pterygoid processes, were identified. The foramina ovalia were documented bilaterally. On the right side, a rare variation of the foramen ovale (FO) was observed (Fig. 2). Endocranially, a complete intraforaminal spur of 3.72/1.60 mm passed infero-laterally from the sphenoidal body to the greater wing and completely separated it into a narrower anterior part of 2.25/3.50 mm (sagittal/transverse diameters) and a larger oblique posterior part, of 8.66/4.60 mm (longitudinal/transverse diameters). Exocranially, the FO appeared reniform (kidney-shaped) in the infratemporal roof, and had 11.07/4.43 mm. A right foramen of Vesalius was found at the medial end of the FO spur. On the left side, a minute canal corresponding to the foramen of Vesalius was coursing through the greater sphenoidal wing, lateral to the vidian canal and anteromedial to the FO.

**Fig. 3:**
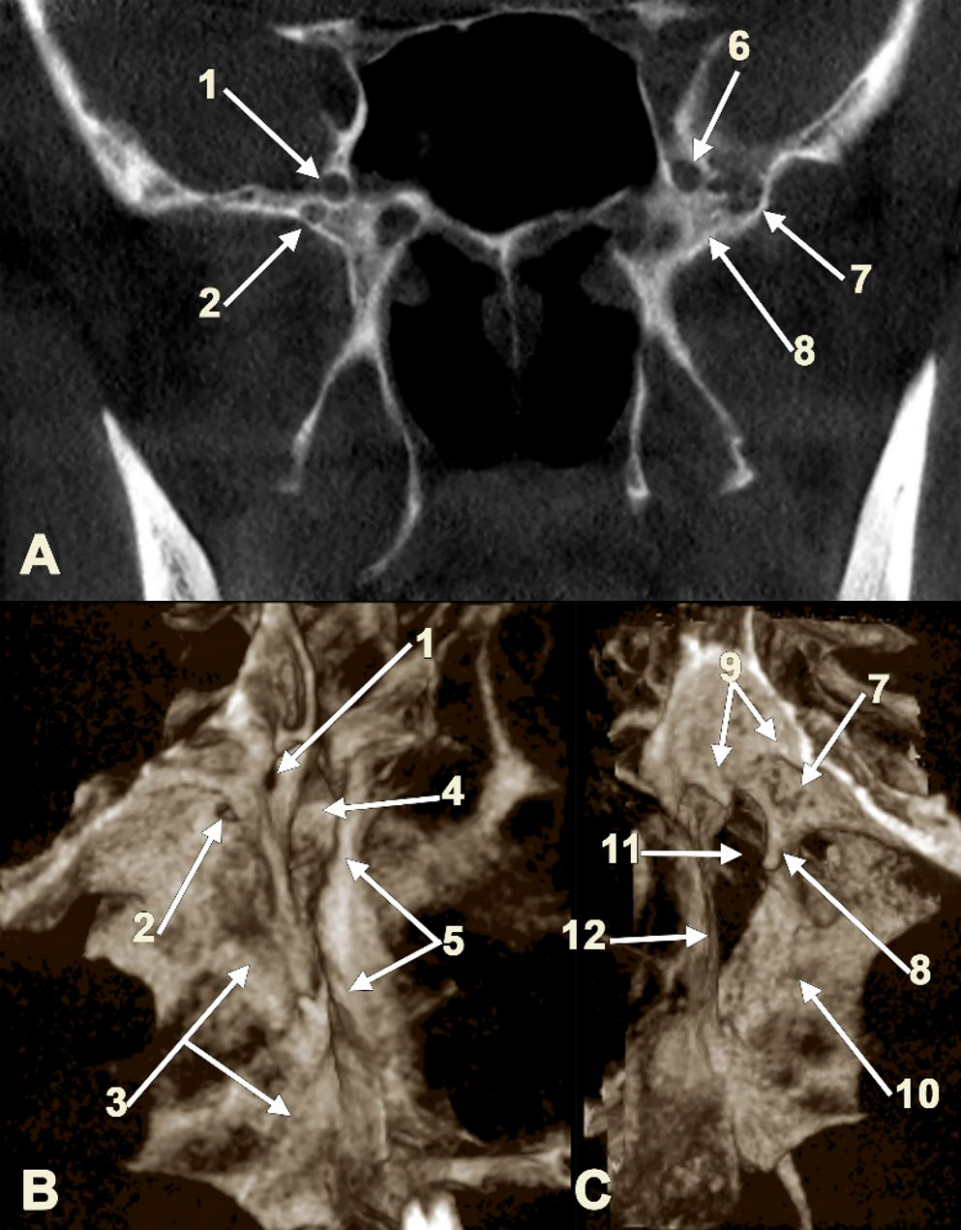
Bilateral multiple foramina rotunda. **A**: coronal slice, viewed anteriorly. **B**: Three-dimensional rendering, right side, antero-lateral view. C. Three-dimensional rendering, left side, postero-lateral view. Right side: (1) foramen rotundum; (2) accessory foramen rotundum (lateral rotundal canal); (3) lateral pterygoid plate; (4) pterygopalatine fossa; (5) posterior wall of the maxillary sinus. Left side: (6) foramen rotundum; (7) cribriform alar zone; (8) sphenoidal tubercle; (9) infratemporal ridge; (10) lateral pterygoid plate; 11. pterygopalatine fossa; 12. posterior wall of the maxillary sinus

On both sides multiple foramina rotunda were found (Fig. 3). The right foramen rotundum appeared double. The main orifice was supero-medially to the accessory one. It had 3.0/3.5 mm (height/width) and corresponded to a 4.91 mm long canal of the greater wing, directed anteriorly, coursing from the middle cranial fossa to the pterygopalatine fossa. The accessory foramen was infero-lateral to the main one, and had 2.06/3.16 mm (height/width). It corresponded to a 3.25 mm long canal, directed antero-inferiorly to open into the infratemporal roof at the junction between the greater sphenoidal wing and the pterygoid root (base). We regarded it as a lateral rotundal canal. On the left side, we found a main foramen rotundum, of 3.61/2.99 mm (height/width), corresponding to a canal long of 3.91 mm that opened anteriorly into the pterygopalatine fossa. Immediately infero-lateral to that main foramen rotundum, an alar area with multiple accessory small foramina (4–5) was documented, and as they opened inferiorly in the infratemporal roof, that alar area was named cribriform alar zone. On the exocranial side, the infratemporal ridge of the greater wing was identified and postero-infero-medially to it was found projected a sharp, inferiorly directed, sphenoidal tubercle of 5.59 mm. Between the attached base of the sphenoidal tubercle and the infratemporal ridge of the greater wing was identified the exocranial side of the cribrifom alar zone. As on both sides the accessory foramina rotunda were opened inferiorly just above the superior (sphenoidal) head of the respective lateral pterygoid muscle, we considered them as foramina of emissary veins that connected inferiorly to the pterygoid venous plexus.

### Case report #2: Inferior rotundal canal

**Fig. 4:**
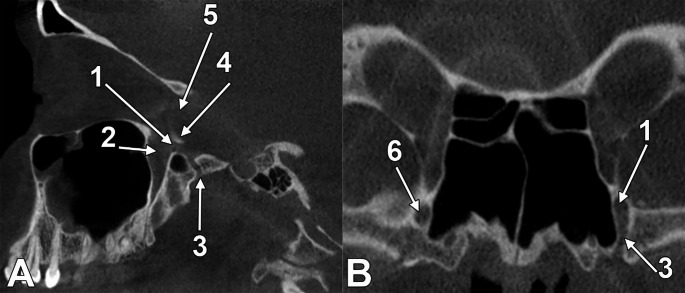
Sagittal (**A**, lateral view) and coronal (**B**, anterior view) slices through the left foramen rotundum and inferior rotundal canal in Case #2. (1) left foramen rotundum; (2) pterygopalatine fossa; (3) inferior rotundal canal; (4) maxillary strut; (5) superior orbital fissure; (6) right foramen rotundum

In a different, female case, was found a FR variant on the left side (Fig. 4). A canal was found descending from the inferior margin of the endocranial opening of the FR. It was termed, according to the terminology of Ginsberg, inferior rotundal canal. This canal was 1.77 mm large and 7.63 mm long. It was directed infero-laterally and opened on the posterior side of the pterygoid root, externally to the scaphoid fossa and tensor veli palatini and superiorly to the pterygoid fossa and the medial pterygoid.

## Discussion

The present study measured a mean FRC length of 4.07 ± 1.78 mm across 88 sides from 45 adult patients, with a range of 0.75–9.12 mm and a coefficient of variation (CV) of 43.8%. The measurement of a distinct, osseous canal length in all examined specimens — rather than a simple aperture — directly supports reconceptualising this structure as the foramen rotundum canal (FRC). The high CV indicates that the FRC is not a dimensionally stable structure: approximately one-third of sides fell below 3 mm and one-third exceeded 5 mm, with a continuous distribution between these extremes. A statistically significant left-sided predominance was identified (4.33 ± 1.77 mm vs. 3.81 ± 1.77 mm; *p* = 0.013, Cohen’s d = 0.34). Gender differences were present in the same direction as reported for other craniofacial foramina but did not reach significance (males 4.22 mm vs. females 3.84 mm; *p* = 0.325, Cohen’s d = 0.22). The interobserver ICC of 0.94 confirms that CBCT-based FRC length measurement is reproducible across observers of different experience levels, provided a standardised sagittal protocol is used.

### Anatomic variations of the FR

The present study identified three distinct accessory-channel variants, each consistent with a recognised category. Case #2 yielded an inferior rotundal canal 7.63 mm long and 1.77 mm wide, opening on the posterior surface of the pterygoid root superiorly to the pterygoid fossa and laterally to the tensor veli palatini — a substantially longer example than the 1–3 mm range described by Ginsberg et al. and Sondheimer [11, 20], suggesting that the inferior rotundal canal spectrum extends further than previously reported. Case #1 demonstrated, on the right side, a lateral rotundal canal 3.25 mm long opening into the infratemporal roof, confirming the extracranial aperture that Ginsberg et al. [11] described but did not localise precisely. On the contralateral side, a cribriform alar zone with four to five accessory foramina likewise opened into the infratemporal roof, consistent with the para-rotundum accessory openings described by Mazengenya and Ekpo [17] in 0.98% of South African skulls. All accessory channels in both cases directed toward the infratemporal roof, not toward the pterygopalatine fossa, which distinguishes them from true FRC duplication in which both canals communicate with the pterygopalatine fossa and contain a duplicated V2 segment [18, 22]. The inferior rotundal canal, lateral rotundal canal, cribriform alar zone, para-rotundum unusual foramina, and the sphenopterygoid canal identified by Rusu [19] collectively represent a spectrum of alternative emissary venous pathways from the middle cranial fossa to the pterygoid venous plexus; their recognition is relevant to avoid misinterpretation on preoperative CBCT imaging. These variants are summarised in Table 4.

**Table 4:** Anatomic variations of the foramen rotundum

Variation/Entity	Defining anatomy (Key feature)	Communication/Extracranial opening	Likely transmitted structure(s)/Unctional interpretation	Distinctive notes (incl. “true vs false” duplication context)	Citations
True duplication of the FRC	Two canals both communicating with the pterygopalatine fossa	Both canals reach pterygopalatine fossa	Contains duplicated segment of V2	Should be distinguished from the false duplications	[18, 22]
Persistent foramen lacerum anterius due to absent maxillary strut	The maxillary strut normally separates FR from SOF; failure to form leads to a persistent foramen lacerum anterius	Persistence of a developmental communication at the skull base (between regions normally separated by the strut)	Not specified	Seen in some lower mammals; quite rare in humans	[11]
Inferior rotundal canal	Small (1–3 mm) opening in the floor of FR	Leads to infratemporal fossa or space between pterygoid plates	Emissary veins	Considered a more common FR variant	[8, 11, 20]
Inferior rotundal canal	A 1.77/7.63 mm canal opened on the posterior side of the pterygoid base	Leads in the pterygoid fossa, above the medial pterygoid muscle and laterally to the tensor veli palatini	Emissary vein	Unilateral evidence	Present study (Case #2)
Lateral rotundal canal	Canal opening lateral to FR with inferolateral trajectory	To the infratemporal fossa, extracranial opening not precisely located by Ginsberg et al.	Emissary vein	Defined by Ginsberg et al. as a named variant	[11]
Lateral rotundal canal	Lateral rotundal canal (as above)	Extracranial opening identified in the infratemporal roof	Emissary vein	Reported distinctively from the contralateral alar cribriform zone	Present study (Case #1)
Unusual foramen adjacent to FR (UF)	Accessory foramen adjacent to FR; most often anterolateral (frequently bilateral), rarely posterolateral	In some specimens a short canal directed inferomedially toward the pterygopalatine fossa; in others the canal is blind/shallow	Interpreted as an emissary venous or incomplete venous/diploic channel (not described as transmitting duplicated V2)	Reported in 5/512 skulls (0.98%); important CT/CBCT differential (may mimic pathology); best regarded as a para-rotundum accessory foramen/canal, not a “true duplication” of the FRC	[17]
Alar cribriform zone	Cribriform area of the alar region	Corresponds to the infratemporal roof	Emissary vein	Reported as a contralateral counterpart to a lateral rotundal canal	Present study (Case #1)
Sphenopterygoid canal (SPC) traversing to a pterygoid foramen (PF)	SPC opens superiorly on the lateral side of FR; opens inferiorly at a PF located at the superior end of the posterior margin of the lateral pterygoid plate	Middle fossa region → PF → pterygoid region	Sphenoidal emissary vein draining to the pterygoid plexus	Presented as another alternative emissary venous pathway	[19]

FR = foramen rotundum; FRC = foramen rotundum canal; SOF = superior orbital fissure; V2 = maxillary nerve; UF = unusual foramen adjacent to FR; SPC = sphenopterygoid canal; PF = pterygoid foramen

### Morphometry of the foramen rotundum

The cross-sectional dimensions of the FRC in the present study are broadly comparable to published CT-based data (Table 5). Berlis et al. [5] measured mean FR height of 3.09 ± 0.69 mm and breadth of 3.29 ± 0.63 mm on macerated skulls, with closely matching coronal CT values (3.13 ± 0.56 mm and 3.11 ± 0.78 mm, respectively). Bhattarai et al. [6] reported height 2.41 ± 0.49 mm and width 2.40 ± 0.55 mm by CT, with no significant side or sex differences. The asymptomatic group of Kastamoni et al. [15] yielded length 2.14 ± 0.47 mm and width 2.05 ± 0.48 mm by 3D-CT. These cross-sectional values are systematically smaller than the longitudinal canal length measurements in the present study, as expected given the different anatomical dimension being measured. The methodological heterogeneity in Table 5 — encompassing dry skulls, coronal CT, axial CT, and 3D reconstruction — underscores the need for standardised multiplanar protocols to enable meaningful inter-study comparison of FRC dimensions.

**Table 5:** Morphometric comparison table

Study	Population	Method	Vertical/ Longitudinal (mm)	Transverse/ Width (mm)	Mean Area (mm^2^)	Key Findings
Acar et al. [1]	Turkey	CT (256-slice)	N/A	3.57 ± 0.74 (Avg)	N/A	FR-Midline distance: 17.51 mm.
Bhattarai et al. [6]	Nepal	CT (Coronal)	2.41 ± 0.49 (H)	2.40 ± 0.55 (W)	4.58 ± 1.49	No significant sex-based difference or side-to-side asymmetry.
Ganesh et al. [10]	India	CT (1 mm)	N/A	N/A	N/A	Measured FR distance to midline (Avg: 18.27 mm).
Kastamoni et al. [15]	Turkey	CT (3D)	2.14 ± 0.47 (L)	2.05 ± 0.48 (W)	3.47 ± 1.59	Asymptomatic group; dimensions significantly smaller in TN patients.
Kastamoni et al. [15]	Turkey	CT (3D)	1.78 ± 0.42 (L)	1.77 ± 0.46 (W)	2.86 ± 1.44	TN Group (Painful side); statistically narrower than asymptomatic group.
Karthikeyan et al. [14]	India	Dry Skull	4.01 ± 0.7 (Lg)	3.74 ± 0.6 (T)	N/A	Right side; distance to midline 16.67 mm.
Karthikeyan et al. [14]	India	Dry Skull	4.56 ± 0.8 (Lg)	3.96 ± 0.9 (T)	N/A	Left side; distance to midline 18.53 mm.
Erbagci et al. [9]	Turkey	CT (Coronal)	3.04 × 3.2 (Avg)	N/A	N/A	TN Group; dimensions highly symmetrical.
Erbagci et al. [9]	Turkey	CT (Coronal)	2.4 × 3.2 (Avg)	N/A	N/A	Control Group; no significant difference from TN patients.
Berlis et al. [5]	Germany	Direct (Skull)	3.09 ± 0.69 (H)	3.29 ± 0.63 (B)	N/A	Direct anatomical measurements of 60 macerated skulls.
Berlis et al. [5]	Germany	CT (Coronal)	3.13 ± 0.56 (H)	3.11 ± 0.78 (B)	N/A	Found good correlation (*r* = 0.61–0.69) for FR in coronal planes.
Ginsberg et al. [11]	USA	CT (Axial)	3.55 (Avg)	N/A	N/A	Identified accessory canals (Inferior & Lateral Rotundal Canals).
Unver Dogan et al. [24]	Turkey	Dry Skull	3.11 (L)	3.11 (W)	N/A	Reported FR as consistently symmetrical in most individuals.

(H) = Height; (Lg) = Longitudinal; (L) = Length; (W) = Width; (B) = Breadth; (T) = Transverse. TN: trigeminal neuralgia

### Canal length: validation and comparative morphometry

The mean FRC length of 4.07 ± 1.78 mm obtained here is consistent with the two independent anatomical references available in the literature (Table 6). Ginsberg et al. [11], using high-resolution axial CT in 123 patients, reported a standard FR length of approximately 3.4 mm based on Sondheimer’s [20] original measurements. Unver Dogan et al. [24], measuring maximum canal length on 44 dry skulls and 18 cadavers, reported 4.48 ± 1.15 mm (right) and 4.36 ± 0.66 mm (left) in a Turkish sample. The present CBCT-based mean falls between these two references, consistent with CBCT providing a direct measurement of the full osseous canal. Importantly, the present study’s right-left values (3.81 mm right, 4.33 mm left) mirror the direction and magnitude of Karthikeyan et al.’s [15] dry skull findings (4.01 mm right, 4.56 mm left), providing independent cross-method confirmation of the left-sided predominance. In contrast, Amin et al.’s [2] V2 canal measurement of 12.45 ± 1.60 mm reflects a surgical drilling corridor rather than a morphometric canal length; it exceeds the present values by approximately threefold and is not directly comparable. The convergence of three independent studies around a 3.4–4.5 mm normative range validates the sagittal CBCT measurement protocol and establishes a reference interval applicable across populations and imaging modalities.

**Table 6:** Comparison of foramen rotundum canal LENGTH measurements across studies

Study	Year	Method	Sample	Parameter	Mean (mm)	SD (mm)	Range (mm)
Present study	2026	CBCT	44 pts (88 FR)	Overall	4.07	1.78	0.75–9.12
				Right side	3.81	1.77	0.75–7.52
				Left side	4.33	1.77	1.00-9.12
Ginsberg et al. [11]	1994	HR-CT (1.5 mm)	123 patients	Canal length	3.4	—	—
Amin et al. [2]*	2010	Endoscopic	5 heads + 4 skulls	V2 canal	12.45	1.60	11.0-15.5
Unver Dogan et al. [24]	2014	Direct measurements	44 dry skulls + 18 cadavers	Canal length	4.48/4.36	1.15/0.66	3.93–5.01

SD = standard deviation; HR-CT = high-resolution computed tomography; PPF = pterygopalatine fossa; MCF = middle cranial fossa

*Amin et al. [2]: Their V2 canal represents an operational endoscopic construct (surgical drilling corridor from PPF to MCF), not a standardised morphometric FR definition. Measurement likely extended beyond strict osseous canal boundaries. Small sample size (*n* = 10 sides), mixed material (formalin cadavers + dry skulls), no intra-/interobserver reliability data. Values should not be directly compared with anatomical FR canal length measurements

The observed range of 0.75–9.12 mm and CV of 43.8% substantially exceed those reported by Unver Dogan et al. [24], whose dry skull range was 3.93–5.01 mm. This difference likely reflects genuine population-level biological variability that a dry skull series underestimates due to sample size and probable exclusion of extreme variants. The 5th–95th percentile interval in the present study (1.75–6.87 mm) may serve as a clinically useful reference for distinguishing normal anatomical variation from pathological narrowing in future outcome-based studies.

### The narrowed foramen rotundum

Although the present study did not include a TN cohort, the observed high variability in FRC length is directly relevant to the debate on foraminal narrowing as a contributor to TN pathogenesis (Table 7). The mean FRC length of 4.07 mm in the present normative sample exceeds by approximately twofold the symptomatic-side dimensions reported by Kastamoni et al. [15]; (painful side 1.78 ± 0.42 mm) and Sönmez et al. [21]; (TN 1.49 ± 0.39 mm), suggesting a large morphometric gap between normal and pathological canal length. At the same time, the 26 sides (29.5%) measuring below 3 mm in the present study indicate that a subset of asymptomatic individuals harbours dimensions approaching those of TN patients, consistent with a multifactorial model of nerve compression. Erbagci et al. [9] found no dimensional difference between TN patients and controls, a negative finding that likely reflects the lower discriminative power of single-slice coronal CT compared to 3D reconstruction, and contrasts with the more recent positive associations [15, 21]. Liu et al.’s [16] borderline finding (symptomatic side 2.50 ± 0.40 vs. 2.71 ± 0.45 mm; *p* = 0.09) in NVC-negative TN patients further suggests that foraminal narrowing may be a relevant contributing mechanism specifically in the absence of neurovascular compression. Congenital FR absence represents the extreme end of this morphological spectrum; Choudhri et al. [7] reported bilateral FR absence with trigeminal nerve agenesis and anaesthesia, underscoring the developmental interdependence of FR morphology and nerve status [3]. These data suggest a dimensional continuum in which the degree of FRC narrowing correlates progressively with trigeminal dysfunction; the present normative dataset provides a baseline against which symptomatic cohorts can be compared.

**Table 7:** Comparison of foramen rotundum morphometric studies

Study	Method	Sample	FR dimensions (mm)	Asymmetry/Difference	TN association
Ginsberg et al. [11]	CT (1.5 mm)	123 patients	Length: 3.4	None observed	Not assessed
Erbagci et al. [9]	CT (0.8 mm)	21 TN / 24 controls	TN: R 3.04 × 3.2, L 2.8 × 2.9; Controls: R 2.4 × 3.2, L 2.5 × 3.1	Not significant (*p* > 0.05)	No association
Liu et al. [16]	CT (0.6 mm)	21 TN / 30 controls	TN: pain 2.50 ± 0.40, non-pain 2.71 ± 0.45; Controls: R 2.68 ± 0.38, L 2.56 ± 0.41	Tendency only (*p* = 0.09)	Possible in NVC-negative cases
Kastamoni et al. [15]	3D-CT (1 mm)	19 TN / 158 controls	TN painful: L 1.78 ± 0.42, W 1.77 ± 0.46; Controls: L 2.14 ± 0.47, W 2.05 ± 0.48	Significant (*p* = 0.001 L; *p* = 0.011 W)	Significant association
Sönmez et al. [21]	CT modified planes (1 mm)	34 TN / 34 controls	TN: L 1.49 ± 0.39, W 1.11 ± 0.39; Controls: L 2.38 ± 0.55, W 1.80 ± 0.48	Highly significant (*p* < 0.001)	Strong association
Ismail et al. [13]	CT (0.6 mm)	57 TN / 40 controls	TN: W 0.21–0.26; Controls: W 0.25–0.28 (cm)	Right narrower (ns); right-sided pain 50.9%	Females narrower in TN (*p* < 0.001)

TN: trigeminal neuralgia; L: length; W: width; R: right; NVC: neurovascular compressions

### Surgical landmarks: the vidian canal and skull base triangles

The mean FRC length of 4.07 mm reported here represents a substantial proportion of the corridor between the middle cranial fossa and the pterygopalatine fossa. Acar et al. [1] reported a mean direct FR-to-vidian canal distance of 5.09 ± 1.64 mm, indicating that the FRC occupies the majority of this inter-landmark interval. In endoscopic skull base surgery, the quadrangular space bounded by the FR and the vidian canal defines the safe zone anterior to the internal carotid artery; the present data quantify the length of osseous canal traversed when accessing the middle cranial fossa via the transpterygoid approach. The FR constitutes the apex of the anteromedial (Müllan’s) triangle [23], and the high inter-individual variability documented here (range 0.75–9.12 mm) implies that this apex position is not constant; preoperative CBCT measurement of FRC length may therefore improve surgical planning accuracy.

### Symmetry and laterality of the foramen rotundum

The statistically significant left-sided predominance in FRC length (4.33 vs. 3.81 mm; *p* = 0.013) constitutes the primary laterality finding of this study. The mean absolute asymmetry was 1.31 ± 1.04 mm (relative asymmetry 35.9 ± 29.6%), indicating that side-to-side differences are clinically non-trivial relative to overall canal length. This direction — left longer than right — is corroborated by Karthikeyan et al.’s [15] dry skull measurements (left 4.56 vs. right 4.01 mm) and aligns with the right-sided narrowing reported by Acar et al. [1], although that study’s methodological limitations preclude direct quantitative comparison. By contrast, Erbagci et al. [9] found no significant side asymmetry in either TN patients or controls, and Bhattarai et al. [6] similarly reported no side-to-side difference in cross-sectional dimensions. These discrepant findings may reflect measurement of different anatomical dimensions: canal length asymmetry (present study) can coexist with symmetrical cross-sectional area. Canal length asymmetry should be considered when interpreting unilateral trigeminal symptoms; its potential relevance to TN laterality warrants prospective evaluation in clinical cohorts.

Males exhibited longer FRC measurements than females (4.22 vs. 3.84 mm), consistent in direction with the general pattern of male-larger craniofacial foramina, but the difference was not statistically significant (*p* = 0.325, Cohen’s d = 0.22). The small effect size and the acknowledged limitation that the gender comparison was performed at the side-measurement level suggest that the present data are insufficient to establish a definitive sex effect on FRC length. Acar et al. [1] reported a sex difference in the FR-to-vidian canal vertical distance (females 4.50 vs. males 3.61 mm), suggesting that sex differences in this region may be more prominent in spatial relationships between landmarks than in canal length itself.

### The divided foramen ovale variant

The present case displayed a foramen ovale (FO) that was reniform on its exocranial aspect and fully partitioned endocranially by a complete bony spur into a narrow anterior compartment (2.25 × 3.50 mm) and a larger oblique posterior compartment (8.66 × 4.60 mm). The overall long-axis dimension (11.07 mm) approximates the upper range of FO length reported by Heeralall et al. [12] in a South African sample. Unlike the present case, Heeralall et al. attributed their compartmentalised specimens to an incomplete ridge producing partial confluence rather than full separation; the complete bony partition in the present case more closely resembles a developmental septation. Recognition of divided FO variants on preoperative imaging is relevant for percutaneous procedures targeting the foramen ovale, such as glycerol rhizotomy.

## Conclusions

This study demonstrates that the FR possesses measurable canal-like dimensions (mean length 4.07 mm) with substantial inter-individual variability, supporting its reconceptualization as the FRC. The significant left-side predominance in canal length and the documentation of rare accessory foramina representing alternative emissary venous pathways underscore the morphological complexity of this region. These findings have potential implications for endoscopic skull base surgery, percutaneous trigeminal interventions, and imaging interpretation. Future studies with larger cohorts and reliability assessments are warranted.

## Data Availability

No datasets were generated or analysed during the current study.
